# A Survey of One Hundred Consecutive Malignant Epithelial Lung Tumours

**DOI:** 10.1038/bjc.1958.39

**Published:** 1958-09

**Authors:** P. J. Mullaney


### A SURVEY OF ONE HUNDRED CONSECUTIVE MALIGNANT EPITHELIAL LUNG TUMOURS

P. J. MULLANEY

*From the Department of Pathology, St. Kevin's Hospital, Dublin*

Received for publication July 24, 1958

PULMONARY carcinoma has reached a position of unparalleled importance because of its increased incidence and the general apprehension of an increasingly higher death rate from this disease in years to come. Interest has been shown in this condition since the year 1800, but in 1911 Adler (1912) found only 360 cases of primary carcinoma of the lung in the literature. Katz (1927) found an increase of from 2·09 per cent in 1900–06 to 11·19 per cent in 1925. Figures published by Doll (1953) for England and Wales show that the death rate from carcinoma of the lung increased by 2½ times from 1900 to 1924 and by 14 times in the next 25 years. In the ages of greatest frequency of lung carcinoma in men, the proportion of this cancer to all other cancers was 40 per cent in 1950. It was the most important site of all cancers in men, causing 28 per cent more deaths than carcinoma of the stomach and providing 24 per cent of all cancers. Of all male deaths in 1950, 4 per cent were due to carcinoma of the lung and in the age group 45 to 54 years the figure was 10 per cent. This condition was a more common cause of death in men than hypertension or pulmonary tuberculosis, but was credited with only one-third as many fatalities as coronary disease. According to Steele (1954), carcinoma of the lung in 1954 was second only to carcinoma of the stomach as cause of death in men in the United States of America, and the death rate from it in that country was twelve times greater in 1950 than in 1920. Smetana, Iverson and Swan (1952) state that figures in the literature indicate that lung carcinoma varies from 5 to 28 per cent of all malignant tumours in men. The deaths from respiratory cancer registered in Ireland (26 counties) have increased from 56 in 1925 to 470 in 1956, and the death rate per 100,000 population has risen from 1·9 in 1925 to 17·2 in 1954, i.e. 800 per cent increase over 1925. The corresponding figures for males are 2·5 and 25·2, and for females 1·2 and 8·9.

Until recent years there has been a difference of opinion as to whether the increase has been apparent or real. For many people, improved diagnosis was the cause of the upward trend and though, no doubt, it has played a part in the increasing number of cases registered, it is generally agreed that it is only one factor in the alarming difference between the figures of the 1950's and those of the 1920's. Earlier estimates may have been too small, for several reasons. There was the tendency to class secondary deposits, which were larger than the lung tumour, as the primary, because of its size. Pulmonary tumours were often regarded as secondary deposits. Classification also played a part, as many tumours designated sarcoma would now be listed as carcinomas. Chemotherapy undoubtedly discloses tumours by prolonging lives which, in some cases, would have been lost because of secondary infection before the tumour was discovered. Whatever criticism may be made of the figures of the estimated increase, there is general agreement that the upward trend in the incidence of lung carcinoma is a very real one.

An interesting feature of this condition is that in most published figures there is a much higher incidence in males than in females. The majority of these ratios vary from approximately 4-to-1 to 11-to-1 for male and female. Kreyberg (1952) quotes Wiklund's figures of 1951 in which 91·1 per cent of cases taken from surgical material were in males. Jaffe (1935) also records a high male incidence in his figure for 4500 autopsies performed between 1915–18. In these, 10·73 per cent of all carcinomas were pulmonary and 92 per cent of them were in males. Bueckley *et al.* (1957) state that foreign-born Mexican women in California have a three-fold excess of lung cancer compared with other women in that State during the period 1949–53. The rate for men was comparable to all men. The ratio for lung carcinoma deaths for the total population of California was: males 4·8 to females 1·0; and for Mexican males 2·0 to Mexican females 1·0. In this paper a Mexican is taken as a person with a Spanish surname. Rakower's (1957) figures for Jewish women of European extraction in Israel are also unusual in this respect. He claims that they have the highest lung carcinoma rate for women in the world, with a figure of 10·8 per 100,000 of the population in 1954.

Figures such as those above and the ratio of the various histological types will vary somewhat depending on whether the specimens are taken from autopsies or from surgical material. The latter tends to represent the younger and fairly fit section of the population. Old people do not so often come to operation or biopsy.

In this report of 100 consecutive cases the material, which was received in a routine diagnostic laboratory in Dublin over a period of approximately two years, consisted of 87 surgical specimens; of these 58 were pneumonectomies and lobectomies, and 29 bronchial biopsies. There were 13 autopsies. Of these cases, 92 were males and 8 females. The ages of males varied between 27 and 77 years, and the females between 34 and 70.

There are various ways of classifying tumours of the lung. Some of these, in many opinions, involve too many types. There are those, including Willis (1948), who believe that there is " only one entity, carcinoma of the lung ", and that the various histological types develop from one primitive pleuripotential cell. If this were so, it would not remove the necessity for classifying these growths, as the tendency to develop into predominant specific histological types may determine the biological behaviour of a tumour. Bignall (1958) states that the median time of survival of those with differentiated tumours (i.e. squamous cell and adenocarcinoma) was 11½ months, that 46 per cent survived for longer than one year, and 25 per cent for two years or more. Of the undifferentiated group, half were dead within 7½ months, and the proportions surviving for one and two years were 31 and 7 per cent.

Table I shows some of the systems of histological classification used over the past thirty years, with the names of the authors and the percentage figures of histological types recorded by them.

Obviously, it would be an advantage that any scheme classifying lung tumours should contain as few categories as possible and that it should be in universal use.

The histological classification of bronchogenic carcinoma is difficult because of the variation and overlap of cell types. In this paper the cases have been classified as squamous cell, undifferentiated, adenocarcinoma, with " oat " cell as a subgroup of the undifferentiated class because it presents a specific histological picture. The critieria of these categories were as follows :

*Squamous cell.* Keratin in small quantities or forming cell nests, intercellular bridges, a line of demarcation between cells, stratification or whorling. Not all of these characteristics were found together.

*Adenocarcinoma.*—Columnar cells showing well formed acini with or without mucous.

*Undifferentiated.*—No uniform cell pattern.

" *Oat* " *cell.*—Presenting a picture conforming to the traditional description of this tumour.

The placing of a tumour in one of these categories does not imply that the growth was wholly of this type, but was predominantly so.

Table II shows that the incidence in this series is : squamous cell 53 per cent, adenocarcinoma 9 per cent, undifferentiated 38 per cent (this last includes " oat " cell, 17 per cent, as a subgroup).

TABLE I.—*Systems of Histological Classification of Malignant Epithelial Lung Tumours*

| | R. H. Jaffe, U.S.A., 1935 (%) | Frissell *et al.*, U.S.A., 1937 (%) | O'Neal *et al.*, U.S.A., 1957 (%) | D.Aunoy *et al.*, U.S.A., 1939 (%) | A. A. Leibow, U.S.A., 1952 (%) | Smetana *et al.*, U.S.A., 1952 (%) | E. Y. Gledhill, U.S.A., 1953 (%) | C. H. Steele, U.S.A., 1954 (%) | T. Wiklund, Sweden, 1951 (%) | L. Kreyberg, Norway, 1954 (%) | J. R. Bignall, G.B., 1955 (%) | K. F. W.Hinson, G.B., 1958 (%) |
|---|---|---|---|---|---|---|---|---|---|---|---|---|
| Squamous cell . | 28 | 30·7 | 29·2 | 50 | 45–60 | 35 | 47·6 | 45·4 | 62·9 | 56·4 | 56·6 | 56 |
| Adenocarinoma . | 24 | 26 | 20 | — | 9–12 | 22 | 16·1 | 21·3 | 4·6 | 8·2 | 8·6 | 11 |
| Undifferentiated . | 31 | — | — | — | — | 42 | 16·2 | 33·3 | 25·1 | — | 34·8 | 28 |
| Large cell . . | — | — | 34·2 | — | — | — | — | — | — | 5·5 | — | — |
| Small cell . . | — | 15·4 | 16·6 | — | — | — | — | — | — | 29·9 | — | — |
| Carcinoma simplex | 4 | — | — | — | — | — | — | — | — | — | — | — |
| Mucous producing | 3 | — | — | — | — | — | — | — | — | — | — | — |
| Mixed cell . . | — | — | — | — | No figures | — | — | — | — | — | — | — |
| Alveolar . . | — | — | — | — | — | — | 1·4 | — | — | — | — | — |
| Uncertain type . | — | — | — | — | — | — | 2·7 | — | 7·4 | — | — | — |
| Reserve cell . | — | — | — | 28·5 | — | — | — | — | — | — | — | — |
| Columnar cell . | — | — | — | 21·5 | — | — | — | — | — | — | — | — |
| Cuboidal cell . | — | 6·7 | — | — | — | — | — | — | — | — | — | — |
| Polymorphous cell | — | 9·2 | — | — | — | — | — | — | — | — | — | — |
| Basal cell . . | — | 2·2 | — | — | — | — | — | — | — | — | — | — |
| " Oat " cell . | 8 | 9·2 | — | — | — | — | 8 | — | — | — | — | 5 |
| Anaplastic . . | — | — | — | — | 28–46 | — | 6·6 | — | — | — | — | — |
| Transitional cell . | — | — | — | — | — | — | 0·7 | — | — | — | — | — |
| Spindle cell . | — | — | — | — | — | — | 0·7 | — | — | — | — | — |
| Scirrhous . . | 2 | — | — | — | — | — | — | — | — | — | — | — |
| Pleomorphic . | — | — | — | — | — | 1 | — | — | — | — | — | — |

| | |
|---|---|
| Frissell and Knox . . | 46 cases. |
| O'Neal *et al.* . . . | 301 ,, |
| Gledhill . . . . | 149 ,, |
| Steele . . . . | 201 ,, |
| D'Aunoy *et al.* . . . | 74 ,, |

The percentages for the cases of each of the above-mentioned authors were abstracted with an accuracy of approximately ± 0·5 per cent. The percentage figures for the remaining writers are those quoted by themselves.

TABLE II.—*Incidence of Histological Types of Lung Tumours in This Series*

| Type | Age groups 20–30 | 30–40 | 40–50 | 50–60 | 60–70 | 70–80 | Total |
|---|---|---|---|---|---|---|---|
| Squamous cell— | | | | | | | |
| Male . . . | — | 1 | 4 | 23 | 19 | 4 | 53 |
| Female . . | — | — | — | 2 | — | — | |
| Adenocarcinoma— | | | | | | | |
| Male . . . | — | — | — | 3 | 3 | — | 9 |
| Female . . | — | — | — | — | 1 | 2 | |
| Undifferentiated— | | | | | | | |
| Male . . . | — | 1 | 3 | 7 | 8 | 1 | 21 |
| Female . . | — | 1 | — | — | — | — | |
| " Oat " cell— | | | | | | | |
| Male . . . | 1 | — | 2 | 6 | 6 | — | 17 |
| Female . . | — | — | — | 1 | 1 | — | |
| | | | | | | | 100 |

Of these 100 cases, squamous cells were found either alone or with other types of cells in 53 tumours : 38 of these contained keratin with or without cell nests. As might be expected, of the total number, 47 were not composed of a single cell type or pattern. Because of this lack of cellular uniformity in so many of the tumours, it was tempting to employ such terms as " large cell ", " large polygonal cell " and " transitional cell ", etc., in an effort to describe various cytological pictures, but this was avoided because of the desirability of using a simple classification.

The Registrar-General's Report for Ireland for 1954 shows that the male/female ratio for deaths from respiratory cancer was 2·8 to 1. It must be pointed out that in these returns the term respiratory cancer includes cancer of the bronchus, lung, trachea, larynx and mediastinum. In the present survey the figures for the total number are : males 11·5 to females 1 ; but in the surgical cases the ratio is16·4 to 1. As this is a preliminary survey, the first of its kind from Ireland to report the histological pattern in 100 cases of bronchial carcinoma, predominantly surgical in origin, it is not intended to comment at the present time on the divergence between the Registrar-General's figures and those of this report, or to make comparisons with figures for lung carcinoma in other countries.

### SUMMARY

One hundred cases of bronchogenic carcinoma are reviewed : 92 were males and 8 females. The ages varied from 27 to 77 years. The material consisted of 58 pneumonectomy and lobectomy specimens, 29 bronchial biopsies and 13 post-mortems. The incidence per cent of histological types was : squamous cell 53, adenocarcinoma 9, undifferentiated 38 (including " oat " cell, 17). Tumours composed of more than one type of cell were found in 47 cases.

My thanks are due to Professor John W. Harman, to Mr. Keith Shaw and Mr. B. O'Neill for the use of the material in this survey.

### REFERENCES

ADLER, I.—(1912) 'Primary, Malignant Growths of the Lung and Bronchi'. New York (Longmans Green & Co.).
BIGNALL, J. R.—(1955) *Lancet*, ii, 210.—(1958) 'Carcinoma of the Lung'. Edited by J. R. Bignall, Edinburgh. (E. & S. Livingstone.)
BUECKLEY, R., DUNNE, J. E., LINDEN, G. AND BRESLOW, L.—(1957) *Cancer*, **10**, 63.
D'AUNOY, R., PEARSONS, B. AND HALPERT, B.—(1939) *Amer. J. Path.*, **15**, 567.
DOLL. R.—(1953) *Brit. med. J.*, ii, 521.
FRISSELL, L. F. AND KNOX, L. C.—(1937) *Amer. J. Cancer*, **30**, 219.
GLEDHILL, E. Y.—(1953) *Delaware St. med. J.*, **25**, 97.
HINSON, K. F. W.—(1958) 'Carcinoma of the Lung'. Edited by J. R. Bignall. Edinburgh (E. & S. Livingstone).
JAFFE, R. H.—(1935) *J. Lab. clin. Med.*, **20**, 1227.
KATZ, K.—(1927) *Z. Krebsforsch.*, **25**, 368.
KREYBERG, L.—(1952) *Brit. J. Cancer*, **6**, 112.—(1954) *Ibid.*, **8**, 199 and 209.
LEIBOW, A. A.—(1952) 'Tumours of the Lower Respiratory Tract'. Sect. 5, Fascicle 17. Armed Forces Inst. of Path., Washington, D.C.
O'NEAL, R. M., KYU TACK LEE and EDWARDS, D. L.—(1957) *Cancer*, **10**, 1031.
RAKOWER, J.—(1957) *Ibid.*, **10**, 67.
SMETANA, H. F., IVERSON, L. AND SWAN, L. L.—(1952) *Milit. Surg.*, **111**, 335.
STEELE, C. H.—(1954) *Ann. Otol., etc., St. Louis*, **63**, 5.
Vital Statistics Report for 1954. (Published in 1957 by Government Publications, Dublin.)
WILLIS, R. A.—(1948) 'Pathology of Tumours'. London (Butterworth & Co. Ltd.).


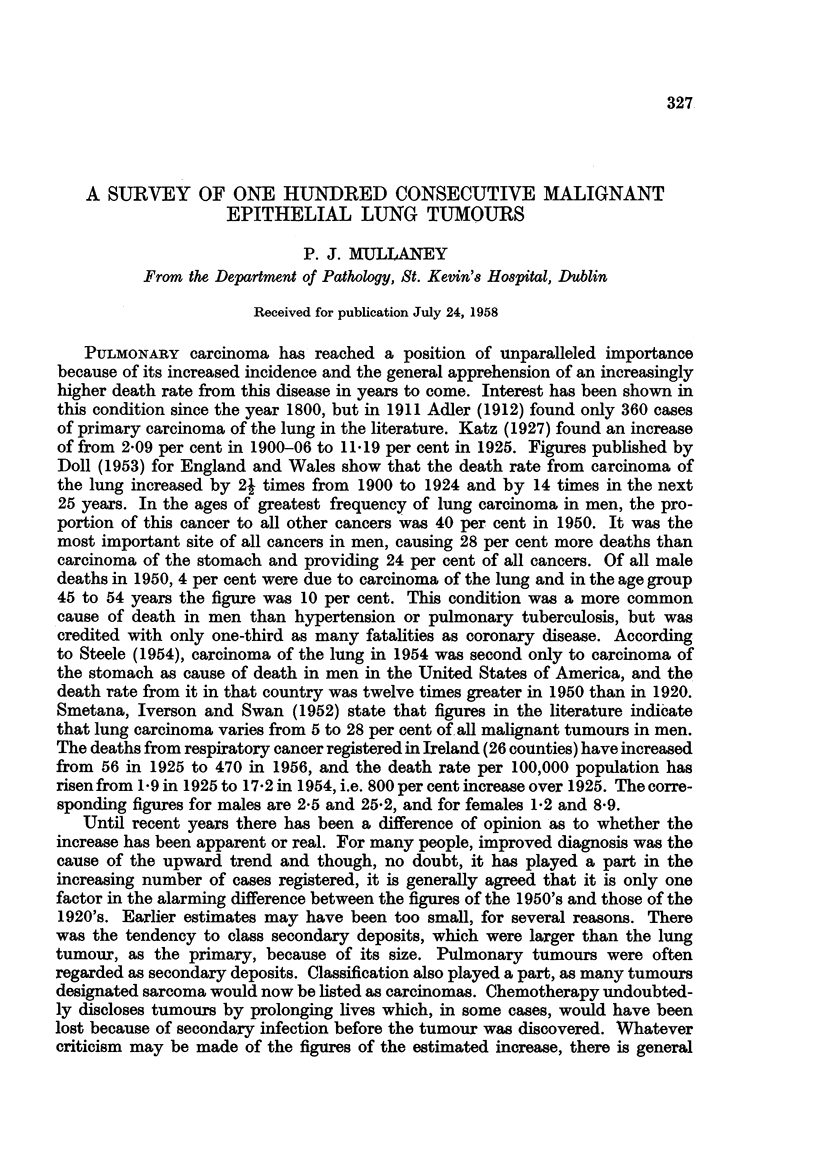

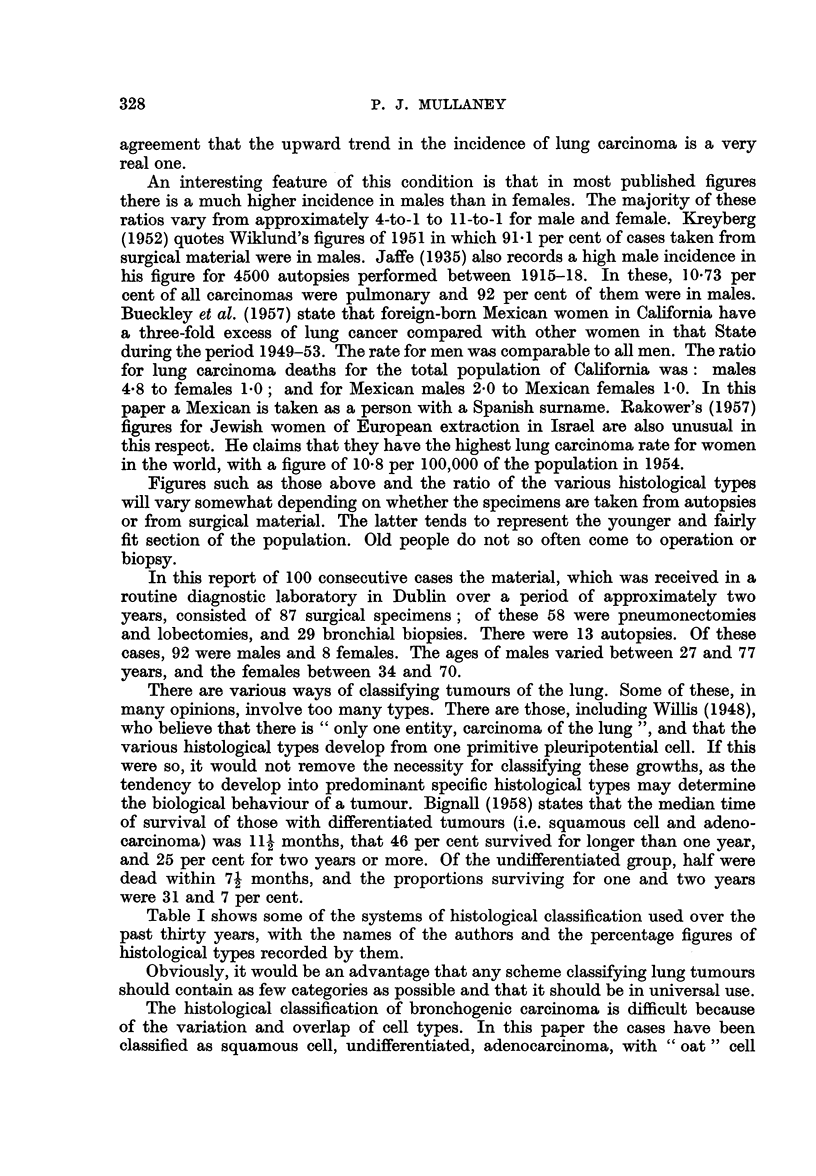

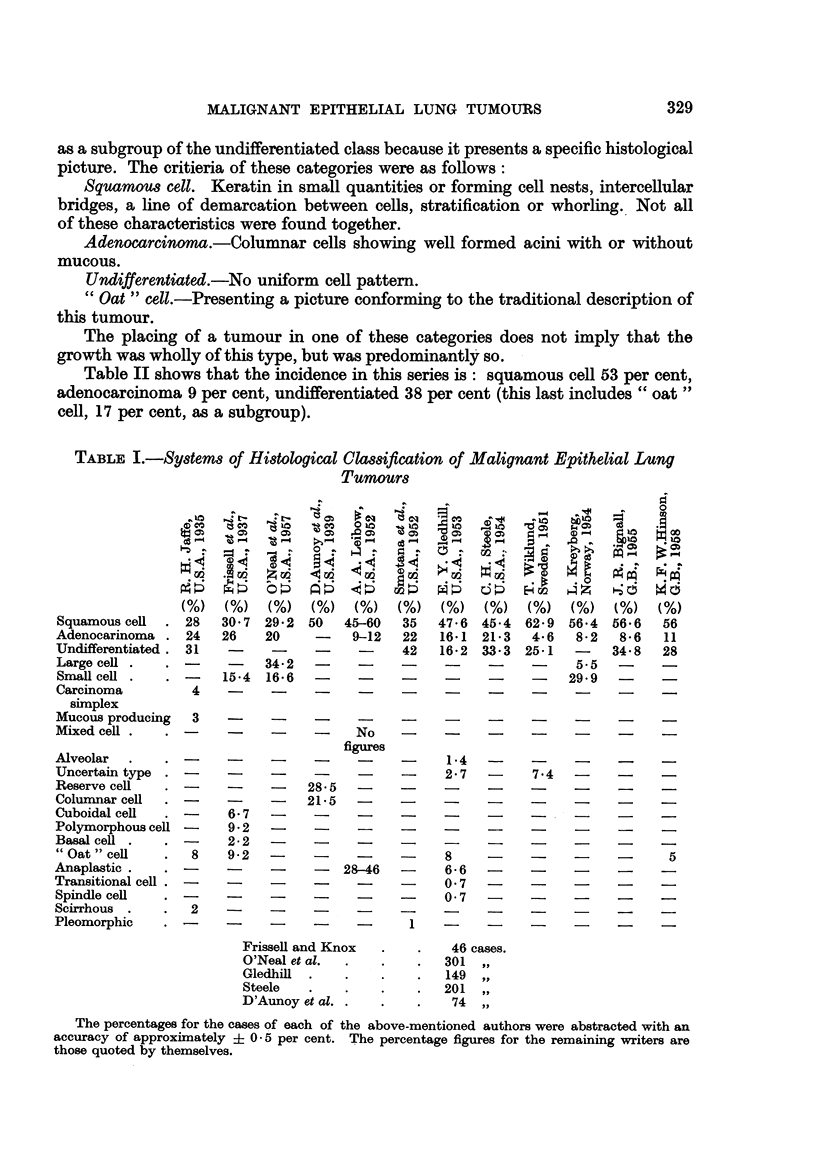

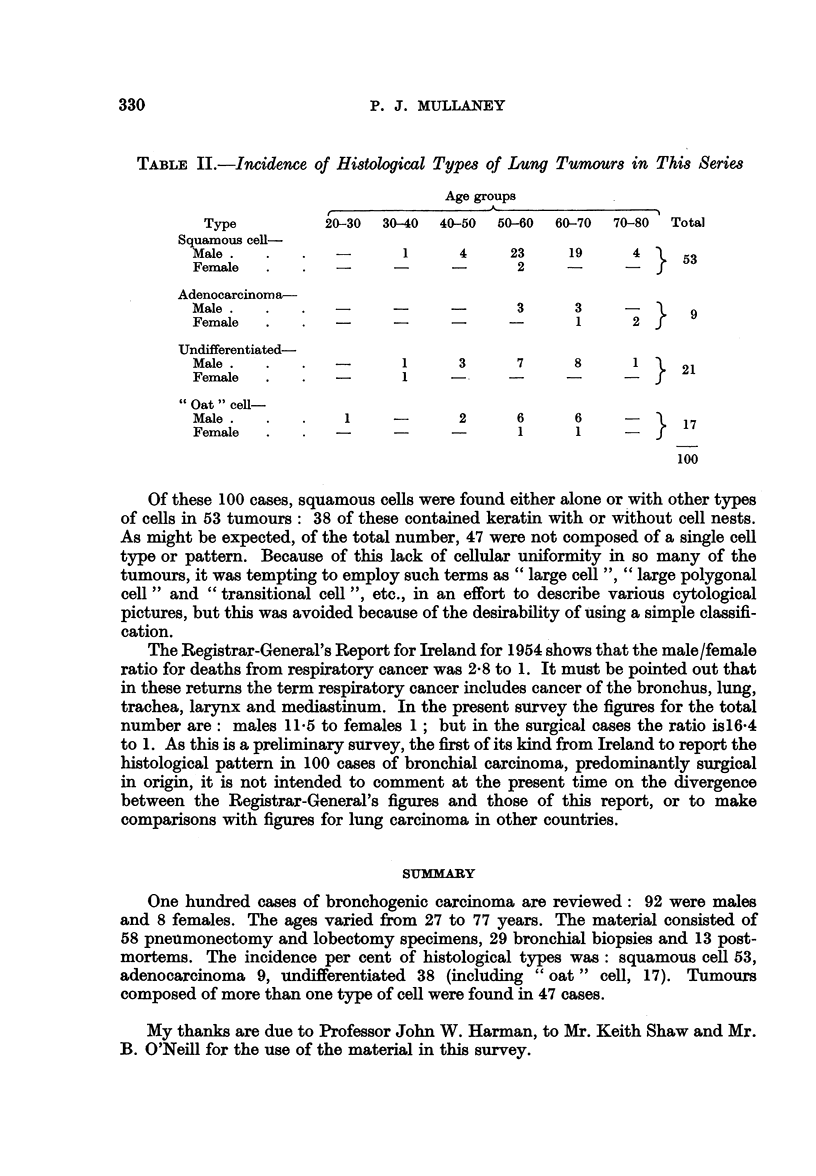

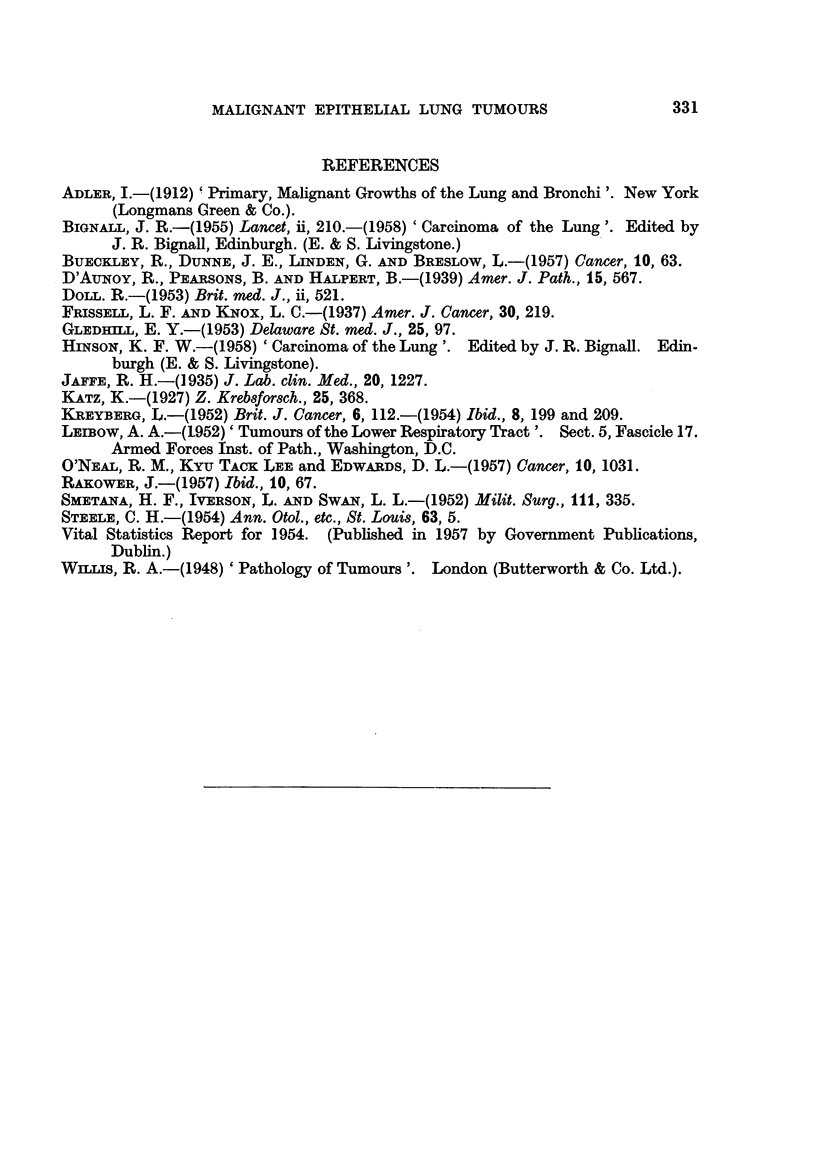


## References

[OCR_00314] D'Aunoy R., Pearson B., Halpert B. (1939). Carcinoma of the lung: An analysis of seventy-four autopsies.. Am J Pathol.

[OCR_00327] KREYBERG L. (1952). One hundred consecutive primary epithelial lung tumours.. Br J Cancer.

[OCR_00333] O'NEAL R. M., LEE K. T., EDWARDS D. L. (1957). Bronchogenic carcinoma; an evaluation from autopsy data, with special reference to incidence, sex ratio, histological type, and accuracy of clinical diagnosis.. Cancer.

[OCR_00336] SMETANA H. F., IVERSON L., SWAN L. L. (1952). Bronchogenic carcinoma; an analysis of 100 autopsy cases.. Mil Surg.

